# Dicyclo­hexyl­ammonium 3,5-dinitro­benzoate

**DOI:** 10.1107/S1600536812027389

**Published:** 2012-06-23

**Authors:** Sohail Saeed, Naghmana Rashid, Rizwan Hussain, Wing-Tak Wong

**Affiliations:** a Department of Chemistry, Research Complex, Allama Iqbal Open Unicversity, Islamabad 44000, Pakistan; b National Engineering & Scientific Commission, PO Box 2801, Islamabad, Pakistan; c Department of Chemistry, The University of Hong Kong, Pokfulam Road, Pokfulam, Hong Kong SAR, People’s Republic of China

## Abstract

The asymmetric unit of the title salt, C_12_H_24_N^+^·C_7_H_3_N_2_O_6_^−^, contains two cations and two anions. In the crystal, the cations and anions are connected by N—H⋯O hydrogen bonds, forming a 12-membered ring with an *R* ^4^_4_(12) graph-set motif. The center of this 12-membered ring coincides with an inversion centre. π–π stacking is observed between parallel benzene rings [centroid–centriod distance = 3.771 (2) Å].

## Related literature

For background to *N*-substituted benzamides, see: Saeed *et al.* (2011*a* ▶,*b* ▶). For the structure of a related 3,5-dinitro­benzamide, see: Saeed *et al.* (2012 ▶).
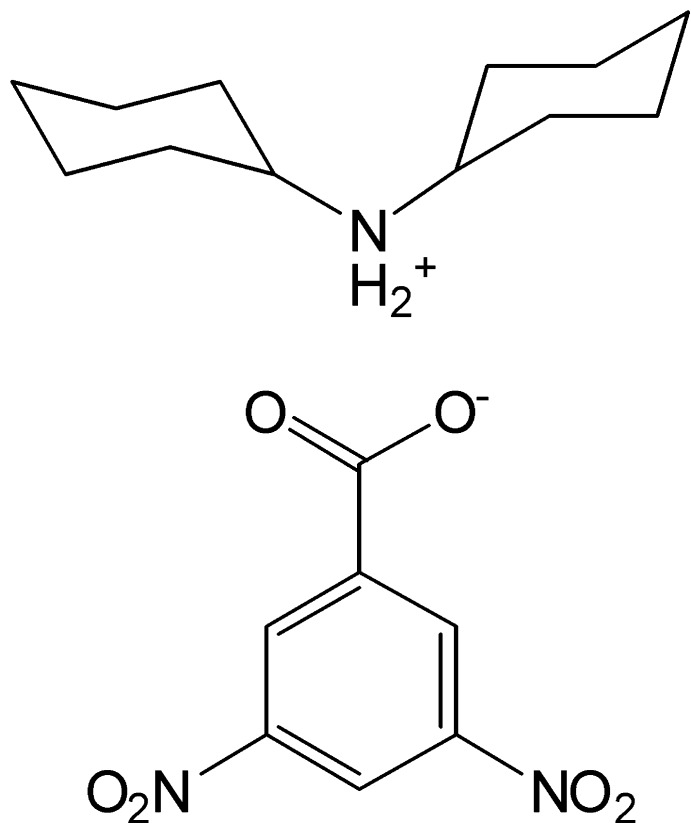


## Experimental

### 

#### Crystal data

C_12_H_24_N^+^·C_7_H_3_N_2_O_6_^−^
*M* *_r_* = 393.44
Triclinic, 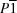

*a* = 9.564 (3) Å
*b* = 10.722 (3) Å
*c* = 20.805 (6) Å
α = 87.294 (5)°
β = 83.226 (5)°
γ = 74.991 (5)°
*V* = 2045.9 (10) Å^3^
*Z* = 4
Mo *K*α radiation
μ = 0.10 mm^−1^
*T* = 296 K
0.55 × 0.26 × 0.08 mm

#### Data collection

Bruker SMART 1000 CCD diffractometer
Absorption correction: multi-scan (*SADABS*; Bruker, 2001 ▶) *T*_min_ = 0.949, *T*_max_ = 0.992
11363 measured reflections
7054 independent reflections
3055 reflections with *I* > 2σ(*I*)
*R*_int_ = 0.032

#### Refinement

*R*[*F* ^2^ > 2σ(*F* ^2^)] = 0.054
*wR*(*F* ^2^) = 0.159
*S* = 1.01
7054 reflections
521 parameters
7 restraints
H atoms treated by a mixture of independent and constrained refinement
Δρ_max_ = 0.22 e Å^−3^
Δρ_min_ = −0.22 e Å^−3^

### 

Data collection: *SMART* (Bruker, 2007 ▶); cell refinement: *SAINT* (Bruker, 2007 ▶); data reduction: *SAINT*; program(s) used to solve structure: *SHELXS97* (Sheldrick, 2008 ▶); program(s) used to refine structure: *SHELXL97* (Sheldrick, 2008 ▶); molecular graphics: *Mercury* (Macrae *et al.*, 2008 ▶); software used to prepare material for publication: *SHELXL97*.

## Supplementary Material

Crystal structure: contains datablock(s) global, I. DOI: 10.1107/S1600536812027389/xu5566sup1.cif

Structure factors: contains datablock(s) I. DOI: 10.1107/S1600536812027389/xu5566Isup2.hkl

Supplementary material file. DOI: 10.1107/S1600536812027389/xu5566Isup3.cml

Additional supplementary materials:  crystallographic information; 3D view; checkCIF report

## Figures and Tables

**Table 1:** Hydrogen-bond geometry (Å, °)

*D*—H⋯*A*	*D*—H	H⋯*A*	*D*⋯*A*	*D*—H⋯*A*
N5—H1*N*⋯O1	0.90 (1)	1.88 (1)	2.768 (4)	169 (4)
N5—H2*N*⋯O2^i^	0.92 (1)	1.83 (1)	2.746 (4)	173 (3)
N6—H3*N*⋯O8^ii^	0.91 (1)	1.86 (1)	2.760 (4)	171 (3)
N6—H4*N*⋯O7^iii^	0.91 (1)	1.87 (1)	2.772 (4)	174 (4)

Symmetry codes: (i) 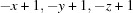; (ii) 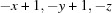; (iii) 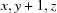.

**Fig. 1.**
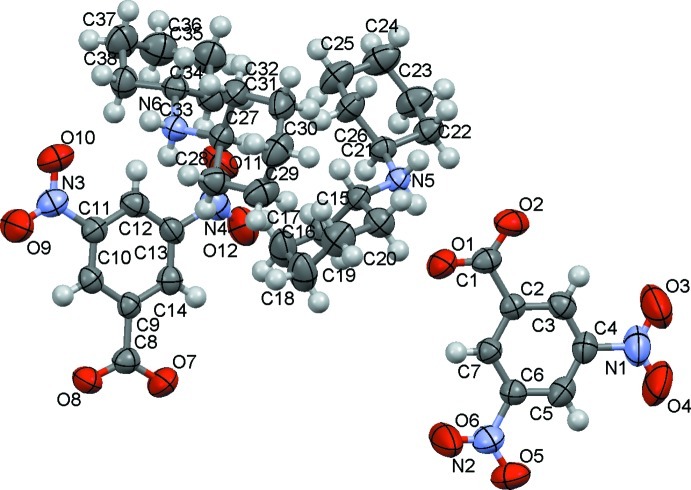
The title compound was shown at 50% probability thermal ellipsoids with the atom numbering scheme.

**Fig. 2.**
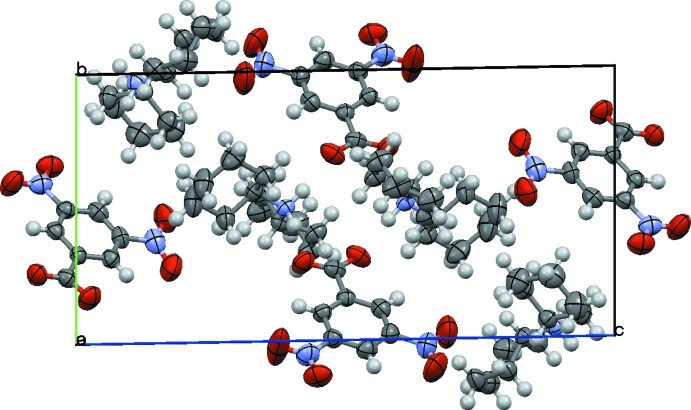
The packing diagram of the unit cell was projected along the a axis and shown at 50% probability thermal ellipsoids.

## supplementary crystallographic information

### Comment

In connection with on-going studies into N-substituted benzamides (Saeed *et al.*, 2011*a*,b), we recently determined the crystal structure of *N*-(4-bromophenyl)-3,5-dinitrobenzamide (Saeed *et al.*, 2012). In this paper we present the crystal structure of the title compound (I), Fig.1. There are two molecules of complex in the asymmetric unit. Each molecule consisted of a dicyclohexylammonium cation and a 3,5-dinitrobenzoate anion. All the cyclohexyl rings in the cation are in chair form. The 3,5-dinitrobenzoate were basically planar. The caboxylate groups / nitro groups make a dihedral angle of 2.0 (3)–16.3 (3)° with the phenyl ring.

Intermolecular H-bonding interactions were observed in the crystal lattice. Two cations and two anions were connected by intermolecular N—H···O H-bonding to form a 12-membered ring, with a graph set assignment, *R*^4^_4_(12). The center of this ring coincide with an inversion centre. π···π interactions also help to stack the 3,5-dinitrobenzoates in the lattice.

### Experimental

To a 250 ml round bottom flask fitted with a condenser was added dicyclohexyl amine (0.01 mol), dichloromethane (15 ml), triethylamine (0.5 ml)with magnetic stirring. 3,5-Dinitrobenzoyl chloride (0.01 mol) was added gradually. The reaction mixture was stirred at room temperature for 1 h and then refluxed for 2 h. The product precipitated out as yellow powder, which was washed three times with water. Recrystallization from ethyl acetate produced the crystals of the title compound.

### Refinement

The structure was solved by direct methods (*SHELXS97*, Sheldrick, 2008) and expanded using Fourier techniques. All non-H atoms were refined anisotropically.

The C-bound H atoms are all placed at geometrical positions with C—H = 0.93, 0.97 and 0.98 Å for phenyl, methylene and methine H-atoms respectively. All C-bound phenyl, methylene and methine H-atoms are refined using riding model with *U*_iso_(H) = 1.2*U*_eq_(Carrier). The N-bound H-atoms are located from difference Fourier map and refined using isotropically with bond distance restraints.

A total of 7 restraints have been used in the refinement, they were bond distances restraints. The N—H bond distances will become too long, 1.00–1.07 Å if not restrained. Thus, the N5—H1N distance was restrained to be 0.87 (1) Å, and the other three N—H distances were restrained to be the same within standard uncertainty of 0.01.

Highest peak is 0.22 at (0.3273, 0.9307, 0.3633) [1.31 Å from O6] Deepest hole is -0.22 at (0.0553, 0.0702, 0.3709) [1.38 Å from O6]

### Crystal data

**Table:**

C_12_H_24_N^+^·C_7_H_3_N_2_O_6_^−^	*Z* = 4
*M**_r_* = 393.44	*F*(000) = 840
Triclinic, *P*1	*D*_x_ = 1.277 Mg m^−^^3^
Hall symbol: -P 1	Mo *K*α radiation, λ = 0.71073 Å
*a* = 9.564 (3) Å	Cell parameters from 11363 reflections
*b* = 10.722 (3) Å	θ = 2.6–25.0°
*c* = 20.805 (6) Å	µ = 0.10 mm^−^^1^
α = 87.294 (5)°	*T* = 296 K
β = 83.226 (5)°	Block, yellow
γ = 74.991 (5)°	0.55 × 0.26 × 0.08 mm
*V* = 2045.9 (10) Å^3^	

### Data collection

**Table:**

Bruker SMART 1000 CCD diffractometer	7054 independent reflections
Radiation source: fine-focus sealed tube	3055 reflections with *I* > 2σ(*I*)
Graphite monochromator	*R*_int_ = 0.032
ω and φ scans	θ_max_ = 25.0°, θ_min_ = 2.6°
Absorption correction: multi-scan (*SADABS*; Bruker, 2001)	*h* = −11→10
*T*_min_ = 0.949, *T*_max_ = 0.992	*k* = −12→12
11363 measured reflections	*l* = −23→24

### Refinement

**Table:**

Refinement on *F*^2^	Primary atom site location: structure-invariant direct methods
Least-squares matrix: full	Secondary atom site location: difference Fourier map
*R*[*F*^2^ > 2σ(*F*^2^)] = 0.054	Hydrogen site location: inferred from neighbouring sites
*wR*(*F*^2^) = 0.159	H atoms treated by a mixture of independent and constrained refinement
*S* = 1.01	*w* = 1/[σ^2^(*F*_o_^2^) + (0.0601*P*)^2^ + 0.1988*P*] where *P* = (*F*_o_^2^ + 2*F*_c_^2^)/3
7054 reflections	(Δ/σ)_max_ < 0.001
521 parameters	Δρ_max_ = 0.22 e Å^−^^3^
7 restraints	Δρ_min_ = −0.22 e Å^−^^3^

### Special details

**Table:**

Geometry. All e.s.d.'s (except the e.s.d. in the dihedral angle between two l.s. planes) are estimated using the full covariance matrix. The cell e.s.d.'s are taken into account individually in the estimation of e.s.d.'s in distances, angles and torsion angles; correlations between e.s.d.'s in cell parameters are only used when they are defined by crystal symmetry. An approximate (isotropic) treatment of cell e.s.d.'s is used for estimating e.s.d.'s involving l.s. planes.
Refinement. Refinement of *F*^2^ against ALL reflections. The weighted *R*-factor *wR* and goodness of fit *S* are based on *F*^2^, conventional *R*-factors *R* are based on *F*, with *F* set to zero for negative *F*^2^. The threshold expression of *F*^2^ > σ(*F*^2^) is used only for calculating *R*-factors(gt) *etc*. and is not relevant to the choice of reflections for refinement. *R*-factors based on *F*^2^ are statistically about twice as large as those based on *F*, and *R*- factors based on ALL data will be even larger.The structure was solved by direct methods (*SHELXS97*, Sheldrick, 2008) and expanded using Fourier techniques. All non-H atoms were refined anisotropically.The C-bound H atoms are all placed at geometrical positions with C—H = 0.93, 0.97 and 0.98 Å for phenyl, methylene and methine H-atoms respectively. All C-bound phenyl, methylene and methine H-atoms are refined using riding model with *U*_iso_(H) = 1.2*U*_eq_(Carrier). The N-bound H-atoms are located from difference Fourier map and refined using isotropically with bond distance restraints.A total of 7 restraints have been used in the refinement, they were bond distances restraints. The N—H bond distances will become too long, 1.00–1.07 (1) Å if not restrained. Thus, the N5—H1N distance was restrained to be 0.87 (1) Å, and the other three N—H distances were restrained to be the same as it within standard uncertainty of 0.01.Highest peak is 0.22 at (0.3273, 0.9307, 0.3633) [1.31 Å from O6] Deepest hole is -0.22 at (0.0553, 0.0702, 0.3709) [1.38 Å from O6]

### Fractional atomic coordinates and isotropic or equivalent isotropic displacement parameters (Å^2^)

**Table:**

	*x*	*y*	*z*	*U*_iso_*/*U*_eq_	
O1	0.4569 (3)	0.2895 (2)	0.42136 (14)	0.0834 (8)	
O2	0.4924 (3)	0.3093 (2)	0.52401 (12)	0.0834 (8)	
O3	0.3366 (5)	0.0233 (4)	0.68820 (16)	0.1603 (17)	
O4	0.2136 (5)	−0.1073 (4)	0.66696 (16)	0.1425 (14)	
O5	0.1046 (3)	−0.1312 (3)	0.44866 (14)	0.0983 (9)	
O6	0.1860 (6)	−0.0206 (4)	0.37519 (18)	0.195 (2)	
O7	0.5031 (3)	0.1717 (3)	0.02474 (13)	0.0906 (8)	
O8	0.5089 (3)	0.2613 (2)	−0.07304 (12)	0.0775 (7)	
O9	0.7548 (4)	0.6023 (3)	−0.11953 (16)	0.1102 (10)	
O10	0.8711 (3)	0.6612 (3)	−0.04920 (13)	0.0908 (8)	
O11	0.8652 (3)	0.4624 (3)	0.16503 (13)	0.1105 (10)	
O12	0.8101 (3)	0.2796 (3)	0.17632 (15)	0.1065 (10)	
N1	0.2786 (5)	−0.0255 (4)	0.65123 (19)	0.1057 (12)	
N2	0.1713 (4)	−0.0526 (3)	0.4302 (2)	0.0871 (10)	
N3	0.7952 (3)	0.5959 (3)	−0.06621 (18)	0.0739 (9)	
N4	0.8184 (4)	0.3779 (4)	0.14607 (17)	0.0831 (10)	
N5	0.5809 (3)	0.4935 (3)	0.38884 (13)	0.0530 (7)	
H1N	0.542 (4)	0.429 (3)	0.4046 (18)	0.117 (16)*	
H2N	0.561 (3)	0.555 (2)	0.4203 (11)	0.075 (11)*	
N6	0.5428 (3)	0.9642 (3)	0.11129 (14)	0.0582 (7)	
H3N	0.533 (3)	0.8903 (17)	0.0950 (12)	0.057 (9)*	
H4N	0.533 (5)	1.028 (3)	0.0807 (15)	0.130 (19)*	
C1	0.4452 (4)	0.2620 (3)	0.4801 (2)	0.0620 (9)	
C2	0.3678 (3)	0.1568 (3)	0.50103 (16)	0.0513 (8)	
C3	0.3572 (3)	0.1147 (3)	0.56540 (18)	0.0638 (9)	
H3	0.3981	0.1504	0.5961	0.077*	
C4	0.2866 (4)	0.0209 (3)	0.58320 (18)	0.0655 (10)	
C5	0.2253 (4)	−0.0359 (3)	0.53991 (19)	0.0677 (10)	
H5	0.1776	−0.0998	0.5528	0.081*	
C6	0.2377 (3)	0.0062 (3)	0.47683 (18)	0.0588 (9)	
C7	0.3069 (3)	0.1010 (3)	0.45714 (16)	0.0560 (8)	
H7	0.3126	0.1275	0.4139	0.067*	
C8	0.5415 (3)	0.2468 (3)	−0.0166 (2)	0.0597 (9)	
C9	0.6339 (3)	0.3311 (3)	0.00360 (16)	0.0504 (8)	
C10	0.6717 (3)	0.4232 (3)	−0.03878 (16)	0.0547 (9)	
H10	0.6418	0.4333	−0.0801	0.066*	
C11	0.7536 (3)	0.4998 (3)	−0.01956 (17)	0.0555 (9)	
C12	0.8015 (3)	0.4888 (3)	0.04056 (18)	0.0640 (10)	
H12	0.8556	0.5424	0.0530	0.077*	
C13	0.7655 (3)	0.3946 (4)	0.08150 (17)	0.0598 (9)	
C14	0.6834 (3)	0.3167 (3)	0.06412 (15)	0.0581 (9)	
H14	0.6609	0.2540	0.0930	0.070*	
C15	0.5094 (3)	0.5547 (3)	0.33090 (15)	0.0576 (8)	
H15	0.5540	0.6244	0.3149	0.069*	
C16	0.5315 (4)	0.4597 (4)	0.27702 (16)	0.0755 (11)	
H16A	0.4897	0.3888	0.2921	0.091*	
H16B	0.6350	0.4245	0.2650	0.091*	
C17	0.4611 (4)	0.5238 (5)	0.21826 (18)	0.1011 (14)	
H17A	0.4715	0.4596	0.1855	0.121*	
H17B	0.5106	0.5880	0.2002	0.121*	
C18	0.3008 (4)	0.5881 (5)	0.23551 (19)	0.1013 (14)	
H18A	0.2613	0.6353	0.1980	0.122*	
H18B	0.2487	0.5225	0.2474	0.122*	
C19	0.2781 (4)	0.6802 (4)	0.2911 (2)	0.0909 (12)	
H19A	0.1745	0.7144	0.3033	0.109*	
H19B	0.3191	0.7520	0.2772	0.109*	
C20	0.3491 (3)	0.6132 (3)	0.34976 (16)	0.0695 (10)	
H20A	0.3368	0.6753	0.3838	0.083*	
H20B	0.3025	0.5461	0.3662	0.083*	
C21	0.7439 (3)	0.4428 (3)	0.37931 (15)	0.0548 (8)	
H21	0.7677	0.3685	0.3507	0.066*	
C22	0.7968 (3)	0.3965 (3)	0.44451 (17)	0.0687 (10)	
H22A	0.7730	0.4684	0.4739	0.082*	
H22B	0.7483	0.3320	0.4630	0.082*	
C23	0.9601 (4)	0.3389 (4)	0.4364 (2)	0.0853 (12)	
H23A	0.9930	0.3123	0.4784	0.102*	
H23B	0.9826	0.2627	0.4098	0.102*	
C24	1.0406 (4)	0.4339 (4)	0.4053 (2)	0.0937 (13)	
H24A	1.1437	0.3916	0.3976	0.112*	
H24B	1.0288	0.5048	0.4344	0.112*	
C25	0.9841 (4)	0.4861 (4)	0.3417 (2)	0.0897 (12)	
H25A	1.0098	0.4172	0.3105	0.108*	
H25B	1.0311	0.5529	0.3250	0.108*	
C26	0.8197 (3)	0.5419 (3)	0.34877 (18)	0.0717 (10)	
H26A	0.7944	0.6179	0.3755	0.086*	
H26B	0.7875	0.5677	0.3065	0.086*	
C27	0.4283 (3)	1.0006 (3)	0.16811 (15)	0.0568 (9)	
H27	0.4444	0.9304	0.2005	0.068*	
C28	0.2796 (3)	1.0151 (4)	0.14522 (17)	0.0757 (11)	
H28A	0.2771	0.9353	0.1258	0.091*	
H28B	0.2627	1.0832	0.1125	0.091*	
C29	0.1594 (4)	1.0473 (4)	0.20176 (19)	0.0842 (12)	
H29A	0.0654	1.0594	0.1859	0.101*	
H29B	0.1717	0.9758	0.2327	0.101*	
C30	0.1633 (4)	1.1676 (4)	0.23485 (19)	0.0872 (12)	
H30A	0.0921	1.1815	0.2728	0.105*	
H30B	0.1377	1.2413	0.2057	0.105*	
C31	0.3140 (4)	1.1572 (4)	0.25515 (17)	0.0881 (13)	
H31A	0.3331	1.0915	0.2890	0.106*	
H31B	0.3153	1.2388	0.2730	0.106*	
C32	0.4351 (4)	1.1232 (3)	0.19912 (16)	0.0708 (10)	
H32A	0.4236	1.1932	0.1673	0.085*	
H32B	0.5291	1.1113	0.2150	0.085*	
C33	0.6991 (3)	0.9351 (3)	0.12455 (15)	0.0587 (9)	
H33	0.7137	1.0107	0.1449	0.070*	
C34	0.7404 (4)	0.8209 (3)	0.16921 (17)	0.0719 (10)	
H34A	0.7219	0.7460	0.1509	0.086*	
H34B	0.6807	0.8380	0.2105	0.086*	
C35	0.8991 (4)	0.7927 (4)	0.17980 (19)	0.0913 (13)	
H35A	0.9147	0.8634	0.2030	0.110*	
H35B	0.9243	0.7150	0.2064	0.110*	
C36	0.9972 (4)	0.7748 (4)	0.1169 (2)	0.0950 (13)	
H36A	0.9927	0.6962	0.0969	0.114*	
H36B	1.0968	0.7651	0.1258	0.114*	
C37	0.9551 (4)	0.8870 (4)	0.0706 (2)	0.0924 (13)	
H37A	1.0141	0.8676	0.0293	0.111*	
H37B	0.9738	0.9631	0.0875	0.111*	
C38	0.7957 (4)	0.9140 (4)	0.06070 (16)	0.0748 (11)	
H38A	0.7795	0.8418	0.0389	0.090*	
H38B	0.7697	0.9903	0.0332	0.090*	

### Atomic displacement parameters (Å^2^)

**Table:**

	*U*^11^	*U*^22^	*U*^33^	*U*^12^	*U*^13^	*U*^23^
O1	0.103 (2)	0.0813 (18)	0.0796 (19)	−0.0484 (15)	−0.0160 (16)	0.0207 (15)
O2	0.0840 (17)	0.0762 (17)	0.097 (2)	−0.0323 (14)	−0.0008 (15)	−0.0296 (15)
O3	0.264 (5)	0.173 (4)	0.070 (2)	−0.097 (3)	−0.035 (3)	0.007 (2)
O4	0.205 (4)	0.140 (3)	0.091 (3)	−0.078 (3)	0.010 (2)	0.039 (2)
O5	0.102 (2)	0.0819 (19)	0.128 (3)	−0.0511 (17)	−0.0192 (18)	0.0037 (18)
O6	0.377 (7)	0.222 (4)	0.079 (3)	−0.233 (5)	−0.064 (3)	0.039 (3)
O7	0.114 (2)	0.091 (2)	0.083 (2)	−0.0593 (17)	−0.0143 (16)	0.0209 (16)
O8	0.1000 (19)	0.0783 (18)	0.0670 (18)	−0.0398 (14)	−0.0207 (15)	−0.0039 (14)
O9	0.158 (3)	0.105 (2)	0.090 (2)	−0.069 (2)	−0.041 (2)	0.0344 (18)
O10	0.0843 (18)	0.0813 (19)	0.116 (2)	−0.0408 (15)	−0.0056 (16)	0.0001 (16)
O11	0.131 (3)	0.129 (3)	0.085 (2)	−0.043 (2)	−0.0366 (18)	−0.0232 (19)
O12	0.123 (2)	0.122 (3)	0.076 (2)	−0.028 (2)	−0.0306 (18)	0.017 (2)
N1	0.151 (4)	0.105 (3)	0.060 (3)	−0.037 (3)	0.003 (2)	0.007 (2)
N2	0.113 (3)	0.078 (2)	0.085 (3)	−0.050 (2)	−0.017 (2)	0.008 (2)
N3	0.076 (2)	0.064 (2)	0.084 (3)	−0.0236 (17)	−0.007 (2)	0.003 (2)
N4	0.076 (2)	0.102 (3)	0.068 (3)	−0.015 (2)	−0.0082 (19)	−0.015 (2)
N5	0.0605 (18)	0.0503 (18)	0.0502 (18)	−0.0199 (14)	−0.0035 (14)	0.0043 (15)
N6	0.0609 (19)	0.060 (2)	0.057 (2)	−0.0211 (15)	−0.0066 (15)	−0.0062 (18)
C1	0.058 (2)	0.052 (2)	0.077 (3)	−0.0165 (17)	−0.001 (2)	−0.008 (2)
C2	0.0474 (18)	0.0505 (19)	0.054 (2)	−0.0090 (15)	−0.0040 (16)	−0.0023 (17)
C3	0.062 (2)	0.065 (2)	0.063 (3)	−0.0120 (18)	−0.0068 (19)	−0.011 (2)
C4	0.074 (2)	0.064 (2)	0.053 (2)	−0.013 (2)	0.004 (2)	0.001 (2)
C5	0.068 (2)	0.060 (2)	0.073 (3)	−0.0192 (18)	0.010 (2)	0.003 (2)
C6	0.062 (2)	0.058 (2)	0.059 (2)	−0.0211 (18)	−0.0047 (18)	−0.0048 (19)
C7	0.059 (2)	0.053 (2)	0.058 (2)	−0.0184 (17)	−0.0088 (17)	0.0036 (17)
C8	0.056 (2)	0.059 (2)	0.064 (3)	−0.0170 (17)	−0.0011 (19)	−0.008 (2)
C9	0.0509 (19)	0.0506 (19)	0.047 (2)	−0.0106 (15)	0.0012 (16)	−0.0056 (17)
C10	0.0533 (19)	0.053 (2)	0.053 (2)	−0.0070 (16)	−0.0005 (16)	−0.0090 (18)
C11	0.055 (2)	0.051 (2)	0.059 (2)	−0.0124 (16)	−0.0002 (18)	−0.0055 (18)
C12	0.058 (2)	0.067 (2)	0.068 (3)	−0.0154 (18)	−0.0060 (19)	−0.015 (2)
C13	0.055 (2)	0.077 (3)	0.048 (2)	−0.0146 (19)	−0.0071 (17)	−0.010 (2)
C14	0.057 (2)	0.060 (2)	0.054 (2)	−0.0101 (17)	−0.0020 (17)	−0.0016 (17)
C15	0.057 (2)	0.062 (2)	0.057 (2)	−0.0227 (16)	−0.0056 (17)	0.0104 (18)
C16	0.072 (2)	0.096 (3)	0.054 (2)	−0.013 (2)	−0.0071 (19)	−0.005 (2)
C17	0.082 (3)	0.158 (4)	0.059 (3)	−0.024 (3)	−0.011 (2)	0.008 (3)
C18	0.081 (3)	0.150 (4)	0.066 (3)	−0.015 (3)	−0.018 (2)	0.012 (3)
C19	0.072 (3)	0.094 (3)	0.100 (3)	−0.009 (2)	−0.020 (2)	0.022 (3)
C20	0.064 (2)	0.070 (2)	0.072 (3)	−0.0114 (18)	−0.0096 (19)	−0.003 (2)
C21	0.0466 (19)	0.058 (2)	0.059 (2)	−0.0133 (16)	−0.0041 (16)	−0.0018 (17)
C22	0.064 (2)	0.066 (2)	0.078 (3)	−0.0221 (18)	−0.0103 (19)	0.015 (2)
C23	0.063 (2)	0.079 (3)	0.113 (3)	−0.017 (2)	−0.018 (2)	0.018 (2)
C24	0.060 (2)	0.091 (3)	0.131 (4)	−0.024 (2)	−0.015 (3)	0.018 (3)
C25	0.065 (3)	0.091 (3)	0.110 (4)	−0.026 (2)	0.009 (2)	0.011 (3)
C26	0.059 (2)	0.077 (2)	0.080 (3)	−0.0253 (19)	0.0035 (19)	0.010 (2)
C27	0.057 (2)	0.061 (2)	0.054 (2)	−0.0194 (16)	−0.0004 (17)	−0.0006 (17)
C28	0.064 (2)	0.083 (3)	0.081 (3)	−0.0203 (19)	−0.005 (2)	−0.014 (2)
C29	0.066 (2)	0.080 (3)	0.106 (3)	−0.023 (2)	0.002 (2)	−0.005 (2)
C30	0.084 (3)	0.080 (3)	0.091 (3)	−0.022 (2)	0.019 (2)	−0.002 (2)
C31	0.112 (3)	0.087 (3)	0.066 (3)	−0.040 (2)	0.023 (2)	−0.020 (2)
C32	0.082 (2)	0.073 (2)	0.062 (2)	−0.031 (2)	0.000 (2)	−0.0079 (19)
C33	0.061 (2)	0.058 (2)	0.060 (2)	−0.0193 (17)	−0.0108 (18)	−0.0011 (18)
C34	0.071 (2)	0.075 (3)	0.070 (3)	−0.0192 (19)	−0.013 (2)	0.004 (2)
C35	0.070 (3)	0.110 (3)	0.092 (3)	−0.018 (2)	−0.020 (2)	0.012 (3)
C36	0.068 (3)	0.101 (3)	0.111 (4)	−0.012 (2)	−0.012 (3)	−0.006 (3)
C37	0.064 (3)	0.121 (4)	0.089 (3)	−0.022 (2)	0.006 (2)	−0.004 (3)
C38	0.068 (2)	0.091 (3)	0.063 (2)	−0.018 (2)	0.001 (2)	0.001 (2)

### Geometric parameters (Å, º)

**Table:**

O1—C1	1.242 (4)	C19—C20	1.525 (5)
O2—C1	1.247 (4)	C19—H19A	0.9700
O3—N1	1.209 (4)	C19—H19B	0.9700
O4—N1	1.213 (4)	C20—H20A	0.9700
O5—N2	1.206 (4)	C20—H20B	0.9700
O6—N2	1.181 (4)	C21—C26	1.512 (4)
O7—C8	1.238 (4)	C21—C22	1.520 (4)
O8—C8	1.244 (4)	C21—H21	0.9800
O9—N3	1.210 (4)	C22—C23	1.516 (4)
O10—N3	1.220 (3)	C22—H22A	0.9700
O11—N4	1.209 (4)	C22—H22B	0.9700
O12—N4	1.218 (4)	C23—C24	1.509 (5)
N1—C4	1.478 (5)	C23—H23A	0.9700
N2—C6	1.468 (4)	C23—H23B	0.9700
N3—C11	1.479 (4)	C24—C25	1.515 (5)
N4—C13	1.477 (5)	C24—H24A	0.9700
N5—C15	1.496 (4)	C24—H24B	0.9700
N5—C21	1.504 (4)	C25—C26	1.523 (4)
N5—H1N	0.900 (9)	C25—H25A	0.9700
N5—H2N	0.918 (12)	C25—H25B	0.9700
N6—C33	1.502 (4)	C26—H26A	0.9700
N6—C27	1.505 (4)	C26—H26B	0.9700
N6—H3N	0.907 (12)	C27—C32	1.511 (4)
N6—H4N	0.910 (12)	C27—C28	1.521 (4)
C1—C2	1.523 (4)	C27—H27	0.9800
C2—C7	1.375 (4)	C28—C29	1.529 (4)
C2—C3	1.394 (4)	C28—H28A	0.9700
C3—C4	1.365 (5)	C28—H28B	0.9700
C3—H3	0.9300	C29—C30	1.501 (5)
C4—C5	1.373 (5)	C29—H29A	0.9700
C5—C6	1.369 (4)	C29—H29B	0.9700
C5—H5	0.9300	C30—C31	1.524 (5)
C6—C7	1.373 (4)	C30—H30A	0.9700
C7—H7	0.9300	C30—H30B	0.9700
C8—C9	1.520 (4)	C31—C32	1.528 (4)
C9—C10	1.383 (4)	C31—H31A	0.9700
C9—C14	1.385 (4)	C31—H31B	0.9700
C10—C11	1.374 (4)	C32—H32A	0.9700
C10—H10	0.9300	C32—H32B	0.9700
C11—C12	1.372 (4)	C33—C34	1.499 (4)
C12—C13	1.377 (4)	C33—C38	1.517 (4)
C12—H12	0.9300	C33—H33	0.9800
C13—C14	1.372 (4)	C34—C35	1.509 (5)
C14—H14	0.9300	C34—H34A	0.9700
C15—C16	1.510 (4)	C34—H34B	0.9700
C15—C20	1.510 (4)	C35—C36	1.506 (5)
C15—H15	0.9800	C35—H35A	0.9700
C16—C17	1.515 (5)	C35—H35B	0.9700
C16—H16A	0.9700	C36—C37	1.507 (5)
C16—H16B	0.9700	C36—H36A	0.9700
C17—C18	1.516 (5)	C36—H36B	0.9700
C17—H17A	0.9700	C37—C38	1.513 (5)
C17—H17B	0.9700	C37—H37A	0.9700
C18—C19	1.516 (5)	C37—H37B	0.9700
C18—H18A	0.9700	C38—H38A	0.9700
C18—H18B	0.9700	C38—H38B	0.9700
			
O3—N1—O4	124.1 (5)	N5—C21—H21	108.2
O3—N1—C4	116.8 (4)	C26—C21—H21	108.2
O4—N1—C4	119.1 (4)	C22—C21—H21	108.2
O6—N2—O5	122.4 (4)	C23—C22—C21	110.1 (3)
O6—N2—C6	118.0 (4)	C23—C22—H22A	109.6
O5—N2—C6	119.7 (4)	C21—C22—H22A	109.6
O9—N3—O10	124.1 (4)	C23—C22—H22B	109.6
O9—N3—C11	117.7 (3)	C21—C22—H22B	109.6
O10—N3—C11	118.1 (3)	H22A—C22—H22B	108.2
O11—N4—O12	125.1 (4)	C24—C23—C22	111.8 (3)
O11—N4—C13	118.1 (4)	C24—C23—H23A	109.3
O12—N4—C13	116.8 (4)	C22—C23—H23A	109.3
C15—N5—C21	116.5 (3)	C24—C23—H23B	109.3
C15—N5—H1N	110 (3)	C22—C23—H23B	109.3
C21—N5—H1N	109 (3)	H23A—C23—H23B	107.9
C15—N5—H2N	107.7 (19)	C23—C24—C25	111.0 (4)
C21—N5—H2N	106.6 (19)	C23—C24—H24A	109.4
H1N—N5—H2N	107 (3)	C25—C24—H24A	109.4
C33—N6—C27	117.2 (3)	C23—C24—H24B	109.4
C33—N6—H3N	104.9 (18)	C25—C24—H24B	109.4
C27—N6—H3N	107.7 (18)	H24A—C24—H24B	108.0
C33—N6—H4N	105 (3)	C24—C25—C26	112.4 (3)
C27—N6—H4N	111 (3)	C24—C25—H25A	109.1
H3N—N6—H4N	111 (3)	C26—C25—H25A	109.1
O1—C1—O2	127.2 (3)	C24—C25—H25B	109.1
O1—C1—C2	116.8 (3)	C26—C25—H25B	109.1
O2—C1—C2	115.9 (4)	H25A—C25—H25B	107.8
C7—C2—C3	118.3 (3)	C21—C26—C25	110.3 (3)
C7—C2—C1	121.0 (3)	C21—C26—H26A	109.6
C3—C2—C1	120.6 (3)	C25—C26—H26A	109.6
C4—C3—C2	119.7 (3)	C21—C26—H26B	109.6
C4—C3—H3	120.2	C25—C26—H26B	109.6
C2—C3—H3	120.2	H26A—C26—H26B	108.1
C3—C4—C5	122.7 (4)	N6—C27—C32	111.9 (3)
C3—C4—N1	119.4 (4)	N6—C27—C28	108.6 (3)
C5—C4—N1	117.9 (4)	C32—C27—C28	110.7 (3)
C6—C5—C4	116.8 (3)	N6—C27—H27	108.5
C6—C5—H5	121.6	C32—C27—H27	108.5
C4—C5—H5	121.6	C28—C27—H27	108.5
C5—C6—C7	122.2 (3)	C27—C28—C29	110.7 (3)
C5—C6—N2	117.4 (4)	C27—C28—H28A	109.5
C7—C6—N2	120.4 (4)	C29—C28—H28A	109.5
C6—C7—C2	120.3 (3)	C27—C28—H28B	109.5
C6—C7—H7	119.9	C29—C28—H28B	109.5
C2—C7—H7	119.9	H28A—C28—H28B	108.1
O7—C8—O8	125.8 (3)	C30—C29—C28	111.1 (3)
O7—C8—C9	117.5 (3)	C30—C29—H29A	109.4
O8—C8—C9	116.7 (3)	C28—C29—H29A	109.4
C10—C9—C14	118.6 (3)	C30—C29—H29B	109.4
C10—C9—C8	120.3 (3)	C28—C29—H29B	109.4
C14—C9—C8	121.1 (3)	H29A—C29—H29B	108.0
C11—C10—C9	119.6 (3)	C29—C30—C31	111.0 (3)
C11—C10—H10	120.2	C29—C30—H30A	109.4
C9—C10—H10	120.2	C31—C30—H30A	109.4
C12—C11—C10	122.8 (3)	C29—C30—H30B	109.4
C12—C11—N3	118.6 (3)	C31—C30—H30B	109.4
C10—C11—N3	118.6 (3)	H30A—C30—H30B	108.0
C11—C12—C13	116.7 (3)	C30—C31—C32	112.9 (3)
C11—C12—H12	121.7	C30—C31—H31A	109.0
C13—C12—H12	121.7	C32—C31—H31A	109.0
C14—C13—C12	122.2 (3)	C30—C31—H31B	109.0
C14—C13—N4	119.7 (4)	C32—C31—H31B	109.0
C12—C13—N4	118.2 (4)	H31A—C31—H31B	107.8
C13—C14—C9	120.1 (3)	C27—C32—C31	109.6 (3)
C13—C14—H14	119.9	C27—C32—H32A	109.8
C9—C14—H14	119.9	C31—C32—H32A	109.8
N5—C15—C16	111.8 (3)	C27—C32—H32B	109.8
N5—C15—C20	110.1 (3)	C31—C32—H32B	109.8
C16—C15—C20	110.6 (3)	H32A—C32—H32B	108.2
N5—C15—H15	108.0	C34—C33—N6	112.7 (3)
C16—C15—H15	108.0	C34—C33—C38	110.7 (3)
C20—C15—H15	108.0	N6—C33—C38	108.8 (3)
C15—C16—C17	111.1 (3)	C34—C33—H33	108.2
C15—C16—H16A	109.4	N6—C33—H33	108.2
C17—C16—H16A	109.4	C38—C33—H33	108.2
C15—C16—H16B	109.4	C33—C34—C35	111.0 (3)
C17—C16—H16B	109.4	C33—C34—H34A	109.4
H16A—C16—H16B	108.0	C35—C34—H34A	109.4
C16—C17—C18	111.4 (3)	C33—C34—H34B	109.4
C16—C17—H17A	109.3	C35—C34—H34B	109.4
C18—C17—H17A	109.3	H34A—C34—H34B	108.0
C16—C17—H17B	109.3	C36—C35—C34	112.0 (3)
C18—C17—H17B	109.3	C36—C35—H35A	109.2
H17A—C17—H17B	108.0	C34—C35—H35A	109.2
C19—C18—C17	111.3 (3)	C36—C35—H35B	109.2
C19—C18—H18A	109.4	C34—C35—H35B	109.2
C17—C18—H18A	109.4	H35A—C35—H35B	107.9
C19—C18—H18B	109.4	C35—C36—C37	112.1 (3)
C17—C18—H18B	109.4	C35—C36—H36A	109.2
H18A—C18—H18B	108.0	C37—C36—H36A	109.2
C18—C19—C20	111.6 (3)	C35—C36—H36B	109.2
C18—C19—H19A	109.3	C37—C36—H36B	109.2
C20—C19—H19A	109.3	H36A—C36—H36B	107.9
C18—C19—H19B	109.3	C36—C37—C38	110.9 (3)
C20—C19—H19B	109.3	C36—C37—H37A	109.5
H19A—C19—H19B	108.0	C38—C37—H37A	109.5
C15—C20—C19	109.9 (3)	C36—C37—H37B	109.5
C15—C20—H20A	109.7	C38—C37—H37B	109.5
C19—C20—H20A	109.7	H37A—C37—H37B	108.0
C15—C20—H20B	109.7	C37—C38—C33	111.5 (3)
C19—C20—H20B	109.7	C37—C38—H38A	109.3
H20A—C20—H20B	108.2	C33—C38—H38A	109.3
N5—C21—C26	112.7 (3)	C37—C38—H38B	109.3
N5—C21—C22	108.5 (3)	C33—C38—H38B	109.3
C26—C21—C22	110.8 (3)	H38A—C38—H38B	108.0

### Hydrogen-bond geometry (Å, º)

**Table:**

*D*—H···*A*	*D*—H	H···*A*	*D*···*A*	*D*—H···*A*
N5—H1*N*···O1	0.90 (1)	1.88 (1)	2.768 (4)	169 (4)
N5—H1*N*···O2	0.90 (1)	2.79 (3)	3.508 (4)	138 (3)
N5—H2*N*···O2^i^	0.92 (1)	1.83 (1)	2.746 (4)	173 (3)
N6—H3*N*···O8^ii^	0.91 (1)	1.86 (1)	2.760 (4)	171 (3)
N6—H4*N*···O7^iii^	0.91 (1)	1.87 (1)	2.772 (4)	174 (4)

Symmetry codes: (i) −*x*+1, −*y*+1, −*z*+1; (ii) −*x*+1, −*y*+1, −*z*; (iii) *x*, *y*+1, *z*.
